# Nuclear *S100A9* Protein Induces Anti-Inflammatory Gene Expression in Sepsis

**Published:** 2025-02-11

**Authors:** Isatou Bah, Dima Youssef, Mary E. Howell, Zhi Q. Yao, Charles E. McCall, Mohamed El Gazzar

**Affiliations:** 1 Department of Internal Medicine and Center of Excellence for Inflammation, Infectious Dieases and Immunity, East Tennessee State University College of Medicine, Johnson City, TN 37614, United States of America; 2 Department of Internal Medicine, Wake Forest University School of Medicine, Winston-Salem, NC 27157, United States of America

**Keywords:** Sepsis, Myeloid-Derived Suppressor Cells (MDSCs), *S100A9*, Immune suppression

## Abstract

Expansion and accumulation of Myeloid-Derived Suppressor Cells (MDSCs) during sepsis contribute to post-sepsis immunosuppression, as these cells suppress innate and adaptive immunity. We have shown that the proinflammatory *S100A9* protein accumulates in the nucleus of MDSCs during late sepsis in both mice and humans. In this context, nuclear *S100A9* acts as a transcription co-factor to induce the expression of two potent immunosuppressive cytokines, Interleukin-10 (IL-10) and Transforming Growth Factor-β (TGF-β). *S100A9* knockdown in MDSCs from late septic mice and late septic patients significantly reduced IL-10 and TGF-β production upon *ex vivo* stimulation with bacterial lipopolysaccharide. In contrast, ectopic expression of *S100A9* in MDSCs from *S100A9*-deficient mice significantly increased IL-10 and TGF-β production. Chromatin immunoprecipitation revealed that *S100A9* protein binds at the IL-10 and TGF-β promoters. Moreover, co-transfection of *S100A9* expression plasmid with a luciferase reporter gene under the control of IL-10 or TGF-β promoter induced the luciferase gene expression in MDSCs from *S100A9*-deficient mice. Notably, in vivo depletion of long noncoding Ribonucleic Acid (RNA) *Hotairm1*, which induces *S100A9* protein accumulation in MDSCs during late sepsis, reduced IL-10 and TGF-β production *ex vivo*. Since IL-10 and TGF-β enhance sepsis immunosuppression and are associated with worse outcomes, our findings suggest that targeting *S100A9* may mitigate the immunosuppressive effects of MDSCs in late sepsis.

## INTRODUCTION

Sepsis is characterized by a dysregulated host response to infection, which can persist after the inciting infection has resolved [1–3]. The initial, severe inflammatory response of sepsis (or acute sepsis) invokes a feedback response designed to promote survival, limit tissue damage and restore immune homeostasis. However, many sepsis survivors proceed to develop chronic critical illness typified by persistent inflammation, immune suppression and protein catabolism [4–7].

Persistence of this post-sepsis response (i.e., late sepsis) increases the risk of secondary bacterial infections and latent viral reactivation and thus elevates morbidity and mortality [1,8]. While enhanced apoptosis of immune cells, T cell exhaustion and epigenetic reprogramming of neutrophils and monocytes can all contribute to the sepsis-induced immunosuppression, dysregulated myelopoiesis leading to expansion of Myeloid-Derived Suppressor Cells (MDSCs) plays a major role in the development of the chronic critical illness that is observed in late sepsis. MDSCs increase the risk of opportunistic infections in septic patients with chronic critical illness [12].

MDSCs are pathologically activated immature myeloid cells with monocytic and granulocytic cell phenotypes [13,14]. While common inflammatory mediators, such as IL-6, IL-1β and growth factors, such as Granulocyte Macrophage Colony-Stimulating Factor (GM-CSF) and Macrophage Colony-Stimulating Factor (M-CSF), as well as transcription factors, such as Nuclear Factor kappaB (NF-kB) and STAT1/3/6, play a role in MDSC expansion under different inflammatory and infection conditions, reprogramming of these immature myeloid cells into immunosuppressive cells is shaped by their microenvironment [13,17]. We and others have shown that MDSCs increase in mice with late sepsis and in critically ill late septic patients [12,18,19].

In addition, we identified an epigenetic pathway that generates immunosuppressive MDSCs during sepsis. Transcripts of the long noncoding RNA *Hotairm1* are increased in MDSCs during the late phases of sepsis in both mice and humans [20,21]. *Hotairm1* couples with the inflammatory protein *S100A9* and shuttles it from the cytosol to the nucleus during late sepsis [22]. We identified nuclear *S100A9* as an immune repressor switch in MDSCs because *S100a9*-deficient mice do not generate immunosuppressive MDSCs and survive sepsis [23].

We have previously demonstrated that MDSCs from mice with late sepsis produce high amounts of immunosuppressive cytokines such as IL-10 and TGF-β. [18]. These inflammatory mediators disrupt T cell functions and activate Treg cells, thus sustaining sepsis-induced immunosuppression [9,24]. We tested whether the *S100A9* protein promotes the immunosuppressive function of MDSCs in late sepsis. The findings indicate that *S100A9* acts as a transcription co-factor to induce the expression of immunosuppressive cytokines. The results suggest that *S100A9* is part of the repressor pathway that sustains post-sepsis immunosuppression.

## MATERIALS AND METHODS

### Mice

Male C57BL/6 mice aged 8 weeks-10 weeks were purchased from the Jackson Laboratory (Bar Harbor, ME). The *S100A9* knockout mice were described previously [23]. The experiments were approved by the East Tennessee State University Animal Care and Use Committee.

### Polymicrobial sepsis

Sepsis was induced by Cecal Ligation and Puncture (CLP) as previously described [25]. The cecum was punctured twice with a 23-gauge needle and fecal matters were extruded into the abdominal cavity. Mice received 1 ml saline for fluid resuscitation. To establish intra-abdominal infection and approximate the clinical condition of human sepsis [26], mice received subcutaneous antibiotic (imipenem; 25 mg/kg) in 0.9% saline at 8 and 16 h after CLP. The mice were followed for 28 days. These manipulations result in early/acute sepsis and late sepsis phases, with ^~^60%-70% mortality during the late phase [18]. Mice that became moribund during late sepsis (days 6-28) were euthanized and analyzed. Mice experiencing hypothermia (<34°C) or loss of righting reflex were considered moribund [25].

### Patients

The study protocol was approved by the Institutional Review Board at the East Tennessee State University College of Medicine (IRB#:0714.6 s). Study participants included patients 18 years or older admitted to Johnson City Medical Center and Franklin Woods Community Hospital in Johnson City, Tennessee, with sepsis or septic shock. Patients were diagnosed with sepsis or septic shock using the Sepsis-3 definitions established by the 2016 international sepsis definitions conference [2]. Sepsis patients were identified based on documented or suspected infection and ≥ 2 points increase in the Sequential Organ Failure Assessment (SOFA) score. SOFA score is set at zero in patients without preexisting organ dysfunctions, which are determined by PaO2, platelets count, Glasgow Coma Scale score, creatinine and bilirubin levels. Septic shock patients presented with persistent hypotension requiring vasopressors to maintain MAP ≥ 65 mm Hg and had serum lactate >2 mmol/L despite adequate fluid resuscitation. Patients were presented with gram-negative or gram-positive bacterial infection in the urinary tract, circulation and respiratory tract. Patients had at least 1 comorbid condition, such as nephropathy, psoriasis, splenectomy, colon cancer or pulmonary disease. Patients with leukopenia due to chemotherapy, glucocorticoid therapy or Human Immunodeficiency Virus (HIV) infection were excluded. Late septic patients had sepsis for more than six days. Blood specimens were collected on days 6-56 after diagnosis. Blood from healthy controls was supplied by Biological *In Vitro* Technologies (BioIVT; Gray, TN). All participants signed an informed consent.

### Myeloid-derived suppressor cells

Mouse Myeloid-Derived Suppressor Cells (MDSCs) were isolated from bone marrow using the EasySep mouse MDSC isolation kit (Stemcell Technologies, Cambridge, MA). The marrow was flushed out with Roswell Park Memorial Institute-1640 (RPMI-1640) medium (Cytiva, Marlborough, MA) and a single-cell suspension was made by filtering through a 70 μm mesh strainer, followed by erythrocyte lysis and washing with PBS. The cell suspension was incubated with a biotin-coupled antibody cocktail at room temperature for 10 min, followed by incubating with streptavidin-coated magnetic beads for 5 min. The enriched Gr-1^+^*CD11b*^+^ cells were cultured in RPMI-1640 medium with 100 U/ml penicillin, 100 μg/ml streptomycin, 2 mM L-glutamine and 10% fetal bovine serum (R&D Systems, Minneapolis, MN).

Human MDSCs were isolated from Peripheral Blood Mononuclear Cells (BPMCs) using Ficoll-Paque PLUS (G.E. Healthcare Life Sciences, Marlborough, MA). MDSCs were depleted of HLA-DR+ cells using an anti-Human Leukocyte Antigen-DR (anti-HLA-DR) biotinylated antibody (Cat#13-9956-82, eBioscience, San Diego, CA) and anti-biotin microbeads (Miltenyi Biotec, Gaithersburg, MD). Next, the remaining cells were positively selected with biotin-coupled antibodies against Cluster of Differentiation33 (CD33) (Cat#MA1-19522; Invitrogen, Waltham, MA), Cluster of Differentiation11b (*CD11b*) (Cat#130-113-795) and Lectin-like Oxidized low-density lipoprotein receptor-1 (LOX-1) (Cat#130-122-119; both from Milteny Biotec).

### *S100A9* knockdown and ectopic expression

For *S100A9* knockdown, MDSCs were transfected with *S100A9*-specific or control small interfering Ribonucleic acid (siRNA) (Qiagen, Germantown, MD) in HiPerFect reagent (Qiagen) at a 0.5 μM final concentration. The cells were cultured in RPMI-1640 medium for 36 h.

For *S100A9* expression, mouse *S100a9* complementary Deoxyribo Nucleic Acid (cDNA) was cloned in the pEZ-M02 mammalian expression vector downstream of the CMV promoter. An empty pReceiver-M02 vector served as a negative control (GeneCopoeia, Rockville, MD). The plasmid DNA was suspended in HiPerFect reagent at 0.5 μg/ml. The MDSCs were transfected and incubated for 36 h.

### Chromatin Immuno Precipitation (ChIP) assay

ChIP was performed using the ChIP-IT express enzymatic shearing kit according to the manufacturer’s protocol (Active Motif, Carlsbad, CA). Briefly, MDSCs were fixed in 1% formaldehyde in an incomplete culture medium at room temperature for 10 min, to cross-link DNA-protein complexes. The cells were lyzed in a buffer with protease inhibitors on ice for 1 h. The lysate was centrifuged at 5,000 rpm and 4°C for 10 min. The pelleted nuclei were digested in a buffer containing an enzymatic shearing cocktail at 37°C for 10 min. The sheared chromatin was recovered by centrifugation at 15,000 rpm for 10 min at 4°C. Approximately 10 μl of the chromatin was reserved as an input sample. Next, 25 μl of protein G-coated magnetic beads and 5 μg of anti-*S100A9* antibody (Cat#MA1-33972; Invitrogen, Carlsbad, CA) or isotype control antibody were added to 150 μl of the sheared chromatin and then immunoprecipitated at 4°C overnight. The beads were washed and eluted in 50 μl elution buffer. The DNA-protein cross-links were reversed with 50 μl of reverse cross-linking buffer and the samples were incubated at 95°C for 15 min. After treatment with proteinase K at 37°C for 1 h, the DNA was recovered and analyzed by Polymerase Chain Reaction (PCR).

### Real-time polymerase chain reaction

To examine the presence of *S100A9* protein at IL-10 and TGF-β promoters, the ChIPed DNA was amplified by real-time quantitative Polymerase Chain Reaction (qPCR) using QuantiTect SYBR Green PCR Master Mix (Qiagen) and primers designed to amplify a 554 bp DNA fragment of the mouse IL-10 proximal promoter and a 571 bp DNA fragment of the mouse TGF-β proximal promoter. These primers were: IL-10 forward (−504 to −483) 5’-GAAAATCAGCCCTCTCGGGGTT-3’; IL-10 reverse (+50 to +31) 5’-TCTGCAAGGCTGCCTTGTGG-3’; TGF-β forward (−500 to −480) 5’-AGGGCCCACTGTTTGGACTGT-3’; TGF-β reverse (+71 to +53) 5’-TGGCTGTCTGGAGGATCC-3’ (Integrated DNA Technologies, Coralville, IA). The PCR was performed in duplicate in 50 μl volumes. The PCR conditions were: 1 cycle at 95°C for 15 min, 35 cycles at 94°C, 58°C and 72°C for 30 s each and a final cycle at 72°C for 10 min. The values were calculated using the 2-ΔΔCt threshold method and normalized to the input DNA samples and the results are presented as a fold change.

### Western blotting

Protein extracts were resolved onto SDS-10% polyacrylamide gel (Bio-Rad, Hercules, CA), blotted for two hours, and then probed overnight at 4°C with anti-*S100A9* antibody (Cat#sc-58706; Santa Cruz Biotechnology, Dallas, TX). The blot was incubated with HRP-conjugated antibody for 2 h at room temperature and the protein was detected with enhanced chemiluminescence reagent (Thermo Fisher Scientific, Waltham, MA). The protein bands were visualized using the ChemiDoc XRS System (Bio-Rad). The membrane was re-probed with a β-actin antibody (Invitrogen).

### Enzyme Linked Immuno Sorbent Assay (ELISA)

Levels of IL-10 and TGF-β in the culture supernatants were determined using specific ELISA kits (Biolegend, San Diego, CA).

### Luciferase assay

The mouse IL-10 promoter sequence (from −500 to +1) and TGF-β1 promoter sequence (from −500 to +1) were synthesized by PCR and cloned in the pEZX-FR01 dual firefly and Renilla vector upstream of firefly luciferase. The Gr1+*CD11b*+ cells were transfected (^~^2 x 10^6^ cells) with 0.5 μg of plasmid DNA (GeneCopoeia) in HiPerFect reagent (Qiagen) for 48 h. The firefly and Renilla luciferase activities were measured using the dual luciferase assay system (Promega, Madison, WI). The GAPDH-FR01 vector, in which the firefly luciferase gene is controlled by the Glyceraldehyde-3-Phosphate De-Hydrogenase (GAPDH) promoter and the Renilla luciferase gene is controlled by the Simian Virus40 (SV40) promoter, served as a positive control.

### Statistical analysis

Data were analyzed with Microsoft Excel. Values are presented as mean ± S.D. Differences between two groups were determined by a two-tailed student’s t-test and p-values <0.05 are considered significant.

## RESULTS

### *S100A9* protein induces IL-10 and TGF-β expression in MDSCs

*S100A9* protein resides in the nucleus in MDSCs in late sepsis [23]. Late sepsis MDSCs promote sepsis immunosuppression because they produce immunosuppressive mediators. We have previously reported high circulating levels of the immunosuppressive cytokines IL-10 and TGF-β in mice during late sepsis [18] and MDSCs from these mice produced copious amounts of IL-10 and TGF-β upon *ex vivo* stimulation with bacterial Lipopolysaccharide (LPS). Since IL-10 and TGF-β play major roles in sepsis-induced immunosuppression [27] and because *S100A9*-defficient mice do not generate immunosuppressive MDSCs [23], we asked whether *S100A9* protein induces IL-10 and TGF-β expression in MDSCs during late sepsis. We focused on late sepsis because the *S100A9* protein moves from the cytosol to the nucleus in MDSCs [20].

**Figure 1:**
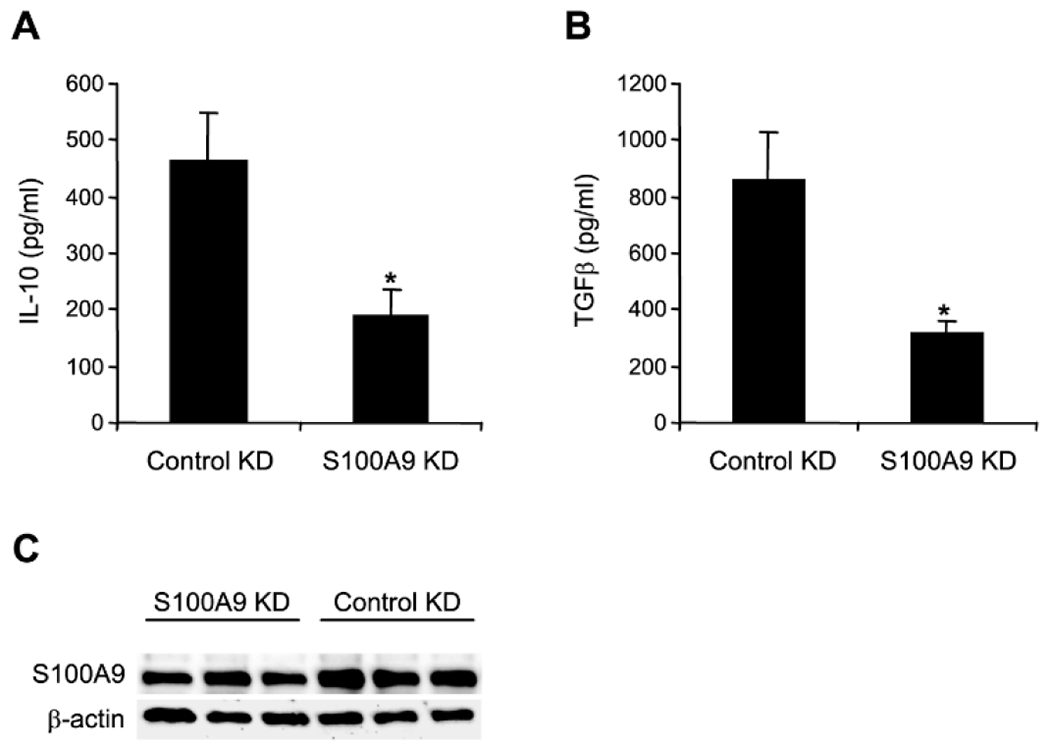
Knockdown of S100A9 in MDSCs from wild-type mice reduces IL-10 and TGF-β expression. Gr1+CD11b+ cells were purified from the bone marrow during the late sepsis phase and transfected with pools of control/scramble or S100A9-specific siRNA. After 36 h, the cells were incubated with 1 μg/ml of bacterial Lipopolysaccharide (LPS, E. coli, serotype 0111:B4) for 12 h, to stimulate IL-10 and TGF-β production. A-B) Levels of IL-10 and TGF-β in the culture supernatants were determined by ELISA. Samples were run in duplicate. The data are the mean ± SD of 5 mice per group. *p<0.05, *vs.* control KD. C) Western blot of S100A9 protein after the knockdown. Results from 3 mice per group are shown. KD, knockdown.

**Figure 2:**
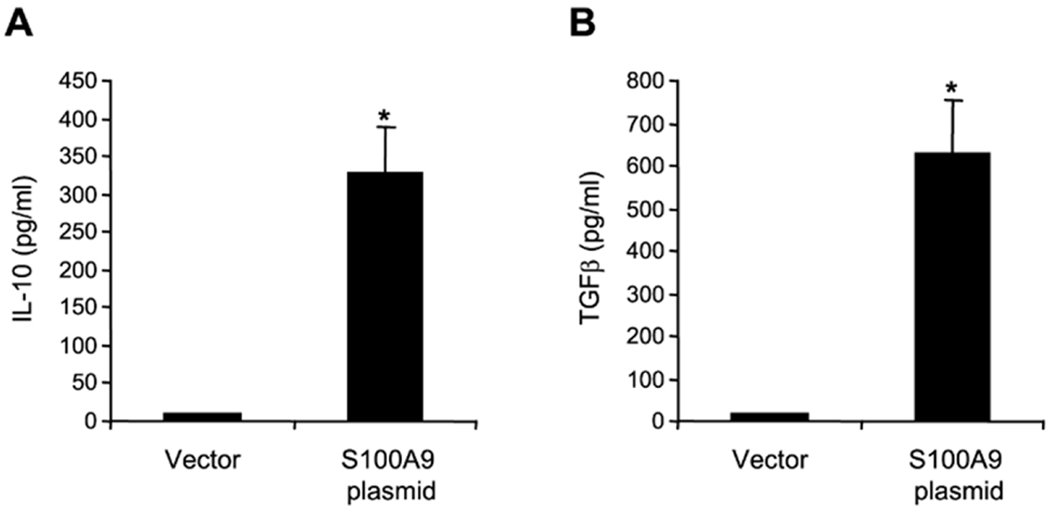
Ectopic expression of S100A9 in MDSCs from S100A9 knockout mice increases IL-10 and (TGF-β) expression. Gr1+CD11b+ cells from late septic mice were transfected with an empty vector or S100A9 expression plasmid. After 36 h, the cells were incubated with 1 μg/ml of bacterial Lipopolysaccharide (LPS) for 12 h. Levels of IL-10 and TGF-β proteins were determined by ELISA. Samples were run in duplicate. The data are the mean ± SD of 5 mice per group. *p<0.05, *vs.* vector.

We first determined IL-10 and TGF-β production by MDSCs following *S100A9* knockdown. As shown in Figure 1, MDSCs with control knockdown produced high levels of IL-10 and TGF-β proteins following LPS for 12 h. *S100A9* knockdown significantly reduced both IL-10 and TGF-β expression. Next, we determined IL-10 and TGF-β production by MDSCs isolated from the *S100A9*-deficient mice during the late sepsis phase. MDSCs lacking *S100A9* produced very small amounts of IL-10 and TGF-β after stimulation with LPS (Figure 2). When these cells were transfected with *S100A9*-expressing plasmid, the levels of IL-10 and TGF-β were increased significantly. These results suggest that *S100A9* promotes IL-10 and TGF-β expression in MDSCs during late sepsis.

### *S100A9* protein binds to IL-10 and TGF-β promoters in MDSCs during late sepsis

**Figure 3:**
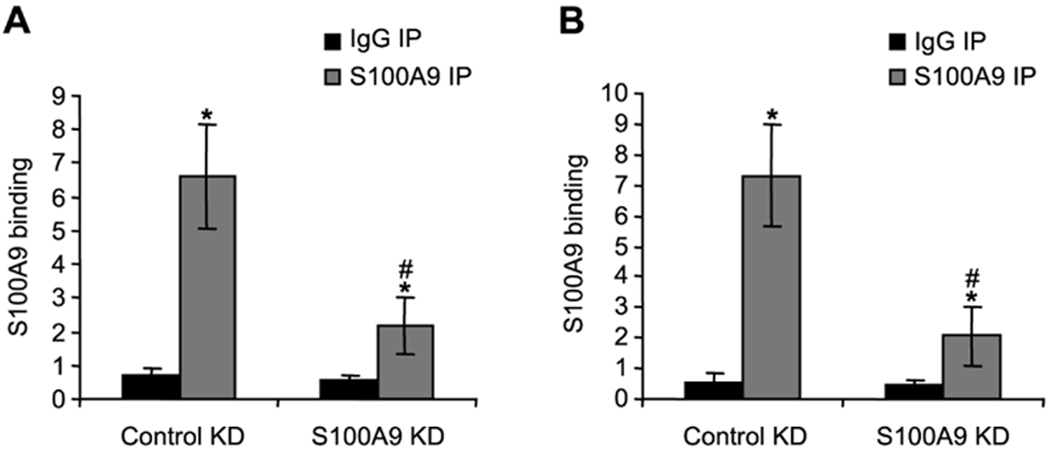
Detection of S100A9 protein binding at IL-10 and TGF-β promoters in MDSCs from wild-type mice. Gr1+CD11b+ cells from late septic mice were transfected with pools of control or S100A9-specific siRNA for 36 h. ChIP assay was performed to detect S100A9 protein binding at IL-10 and TGF-β promoters. The DNA was analyzed by real-time quantitative Polymerase Chain Reaction (qPCR) using primers that amplify DNA sequences spanning ~500bp of the promoter of IL-10 (A) or TGF-β (B). Sample values were normalized to the input DNA (DNA isolated before the immunoprecipitation). The results are the mean ± SD of 5 mice per group. *p<0.05, *vs.* IgG IP; ^#^p<0.05, *vs.* control KD. KD, knockdown.

**Figure 4:**
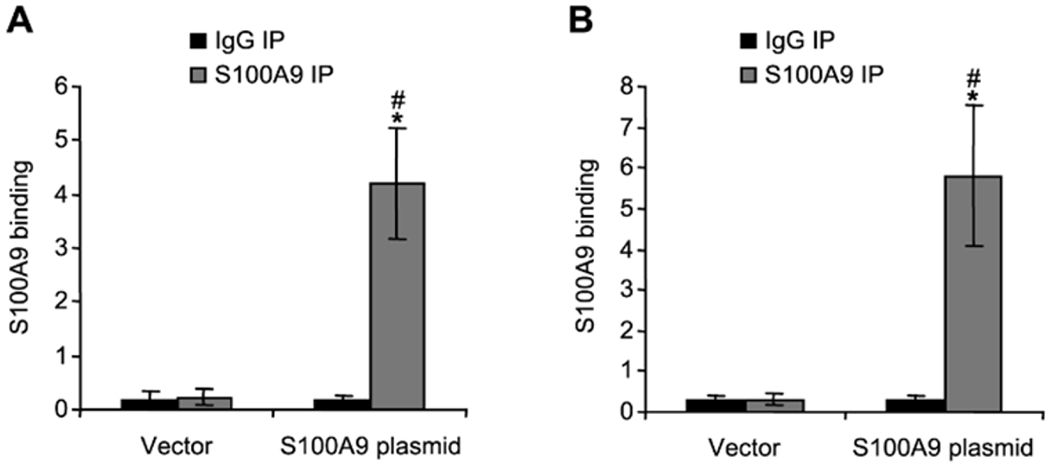
Detection of S100A9 protein binding at IL-10 (A) and TGF-β(B) promoters in MDSCs from S100A9 knockout mice. Gr1+CD11b+ cells from late septic mice were transfected with an empty vector or S100A9 plasmid for 36 h. ChIP assay was performed as described in Figure 3. The results are the mean ± SD of 5 mice per group. *p<0.05, *vs.* IgG IP; #p<0.05, *vs.* vector.

We tested whether *S100A9* protein directly regulates IL-10 and TGF-β expression in MDSCs during the late/protracted phase of sepsis. We performed a ChIP assay using an anti-*S100A9* antibody and chromatin isolated from MDSCs from wild-type mice with late sepsis. The results showed high amounts of *S100A9* protein bound to both IL-10 and TGF-β promoters (Figure 3). *S100A9* protein binding was significantly decreased after *S100A9* knockdown compared to the control knockdown. To confirm *S100A9* binding at the promoters of these cytokine genes, we introduced *S100A9* into MDSCs from *S100A9*-deficient mice undergoing late sepsis response. The cells were transfected with an empty vector or *S100A9* expression plasmid. ChIP and real-time PCR showed high amounts of the ectopically expressed *S100A9* protein at both IL-10 and TGF-β promoters (Figure 4). These results demonstrate that the *S100A9* protein directly targets IL-10 and TGF-β promoters in MDSCs during late sepsis.

### *S100A9* protein can activate reporter gene expression in *S100A9*-deficient MDSCs

**Figure 5:**
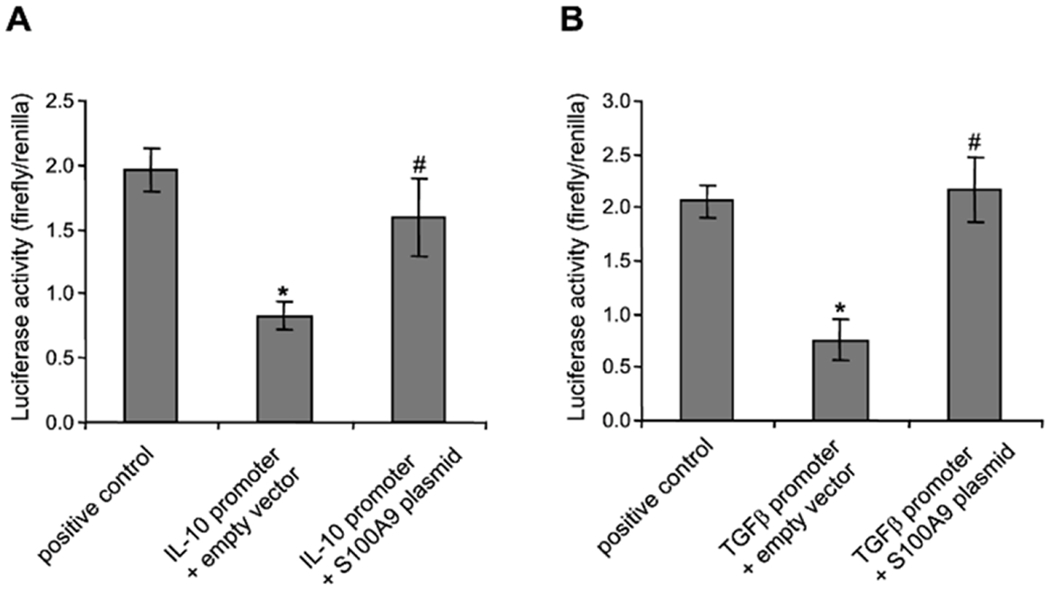
S100A9 protein can activate a luciferase gene driven by IL-10 or TGF-β promoter in MDSCs from S100A9 knockout mice. Gr1+CD11b+ cells from late septic mice were transfected with luciferase constructs containing IL-10 or TGF-β promoter plus empty vector or S100A9 plasmid. After 36 h, the cells were incubated with 1 μg/ml of LPS for 12 h. The cells were harvested for the measurement of firefly and Renilla luciferase activities. The luciferase gene under the control of the GAPDH promoter serves as a positive control for maximum firefly luciferase gene activity. Firefly luciferase values were normalized to Renilla luciferase. The results are the mean ± SD of 5 mice per group. *p<0.05 *vs.* positive control; #p<0.05, *vs.* empty vector.

To functionally examine the effects of *S100A9* protein on IL-10 and TGF-β promoters, we generated luciferase constructs containing 500 bp of IL-10 or TGF-β promoter. The constructs were transfected into MDSCs isolated from *S100A9*-deficient mice undergoing late sepsis. Compared with the positive control construct, where the firefly gene is under the control of the GAPDH promoter, the luciferase activity derived from the luciferase gene under the control of IL-10 or TGF-β promoter was reduced significantly (Figure 5). Notably, ectopic expression of *S100A9* by co-transfection of *S100A9* plasmid resulted in a significant increase in luciferase gene activity compared to co-transfection with the empty vector. These results show that *S100A9* protein can activate the expression of IL-10 or TGF-β promoter-driven luciferase gene in MDSCs.

### *S100A9* protein binds to IL-10 and TGF-β promoters in MDSCs from late septic patients

**Figure 6:**
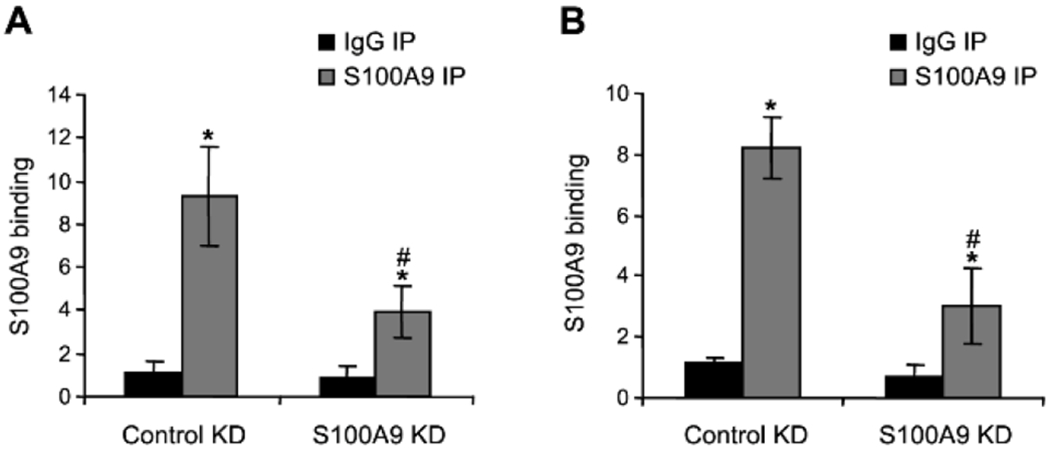
S100A9 binding at IL-10 and TGF-β promoters in MDSCs from late septic patients. CD33+CD11b+LOX1+ cells were isolated from the peripheral blood of late septic patients by positive selection. The CD33+CD11b+LOX1+ cells were transfected with pools of control or S100A9-specific siRNA for 36 h. The binding of S100A9 protein at the IL-10 (A) and TGF-β promoters (B) was detected by ChIP assay. The ChIP DNA was analyzed by quantitative qPCR using primers that amplify DNA sequences spanning ~500 bp of proximal IL-10 or TGF-β promoter. Sample values were normalized to the input DNA. The results are the mean ± SD of 4 patients per group. *p<0.05, *vs.* IgG IP; ^#^p<0.05, *vs.* control KD.

We also examined *S100A9* protein binding at IL-10 and TGF-β promoters in MDSCs isolated from peripheral blood of late septic patients. We have previously shown that *S100A9* protein moves from the cytosol to the nucleus in MDSCs during late sepsis in humans [20]. ChIP assay detected high amounts of *S100A9* protein at the IL-10 promoter (Figure 6A). Knockdown of *S100A9* significantly reduced *S100A9* protein binding at the IL-10 promoter. Almost similar binding patterns were detected at the TGF-β promoter before and after *S100A9* knockdown (Figure 6B).

### *In vivo* depletion of *Hotairm1* inhibits *S100A9* protein binding at IL-10 and TGF-β promoters and reduces cytokines production

**Figure 7:**
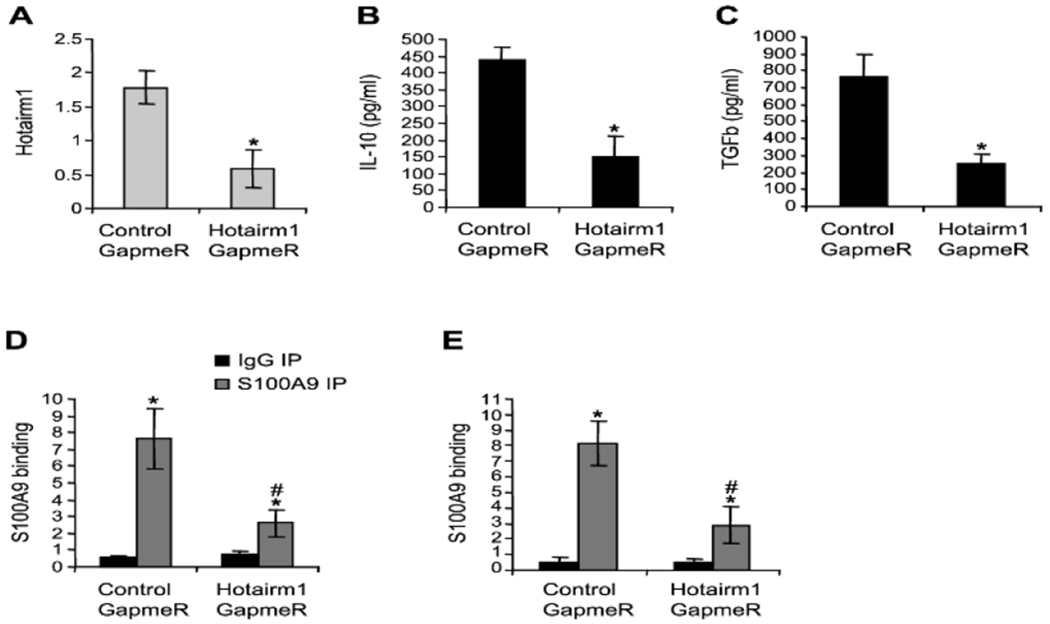
Knockdown of *Hotairm1* in vivo in wild-type mice reduces S100A9 binding at IL-10 and TGF-β promoters and subsequently attenuates cytokine production. Forty-eight hours after CLP, the mice were injected (via tail vein) with antisense oligonucleotides against *Hotairm1* (*Hotairm1* GapmeR) or negative control GapmeR at a dose of 1 ug in 50 ul saline. Gr1+CD11b+ cells were purified from the bone marrow during the late sepsis phase and stimulated with 1 ug/ml of bacterial LPS for 12 h. A: PCR analysis of *Hotairm1* transcripts after the knockdown. B-C: Levels of IL-10 and TGF-β in the culture supernatants were determined by ELISA. The results are the mean ± SD of 5 mice per group. *p<0.05, *vs.* control GapmeR. D-E: Gr1+CD11b+ cells were fixed in formaldehyde and the ChIP assay was performed to detect S100A9 protein binding at IL-10 (D) and TGF-β promoters (E) as described in Figure 3. The results are the mean ± SD of 5 mice per group. *p<0.05, *vs.* IgG IP; ^#^p<0.05, *vs.* control GapmeR.

Levels of noncoding RNA *Hotairm1* are increased significantly in MDSCs in mice and humans during late sepsis [20]. We reported that *Hotairm1* binds to and shuttles *S100A9* to the nucleus in MDSCs during late sepsis [22], suggesting that *Hotairm1* couples with *S100A9* to maintain the immunosuppressive phenotype of MDSCs. We knocked down *Hotairm1* in wild-type mice in vivo by administering chemically modified antisense GapmeR against *Hotairm1* following sepsis induction. We first assessed IL-10 and TGF-β production by MDSCs. MDSCs from late sepsis mice were stimulated with LPS. Forty-eight hours after Cecal Ligation and Puncture (CLP), the mice were injected (via tail vein) with antisense oligonucleotides against *Hotairm1* (*Hotairm1* GapmeR) or negative control GapmeR at a dose of 1 μg in 50 μl saline. Gr1+*CD11b*+ cells were purified from the bone marrow during the late sepsis phase and stimulated with 1 μg/ml of bacterial LPS for 12 h. Figure 7A shows that *Hotairm1* transcripts were significantly reduced after *Hotairm1* knockdown. MDSCs from mice injected with *Hotairm1* GapmeR produced significantly lower amounts of IL-10 and TGF-β compared to mice receiving control GapmeR (Figure 7B–C).

We also examined *S100A9* binding at IL-10 and TGF-β promoters. ChIP assay showed that *Hotairm1* knockdown reduced the amounts of *S100A9* protein at both IL-10 and TGF-β promoters (Figures 7D–7E). These results suggest that *Hotairm1* promotes *S100A9* binding at and activation of IL-10 and TGF-β promoters in MDSCs in late sepsis.

## DISCUSSION

Expansion of MDSCs during late sepsis promotes immunosuppression in mice and humans [12,18,28]. The present study demonstrates that the inflammatory mediator *S100A9* protein enhances the immunosuppressive functions of MDSCs. We found that *S100A9* induces the expression of IL-10 and TGF-β in MDSCs. *S100A9* protein assembled at IL-10 and TGF-β promoters in MDSCs during late sepsis in mice and humans, and *S100A9* could activate a reporter gene controlled by IL-10 or TGF-β promoter. Given the roles of IL-10 and TGF-β in enhancing sepsis-induced immunosuppression [27], these findings suggest that the *S100A9* protein functions as a molecular immune repressor in late sepsis via supporting MDSC suppressive functions.

*S100A9* is well known for its proinflammatory effects, as it amplifies inflammatory responses by promoting phagocyte trafficking and activation and inducing the production of proinflammatory cytokines and reactive oxygen species by various immune cells in many infectious and inflammatory conditions [29–31]. *S100A9* protein functions mainly extracellularly as a secreted mediator of inflammation, but it can also regulate some cellular processes such as cell growth and differentiation by acting as a Ca^+2^ sensor [32,33]. *S100A9* is produced by many immune cells, including monocytes and neutrophils [29]. In the context of MDSCs, we have previously demonstrated that MDSCs secrete copious amounts of *S100A9* during the acute (early) phase of sepsis in mice [23]. As sepsis enters a late phase response, MDSCs lose their ability to secrete *S100A9*, as it becomes dephosphorylated due to binding to *Hotairm1* and moves from the cytosol to the nucleus. Accumulation of *S100A9* protein in the nucleus supports the immunosuppressive function of MDSCs, suggesting that nuclear *S100A9* may act as an immune repressor during late sepsis [20]. In line with this, we find that *S100A9*-deficient mice do not produce immunosuppressive MDSCs in late sepsis [23]. The current study further supports our previous findings that *S100A9* protein promotes the immunosuppressive effects of MDSCs, as the results showed that *S100A9* induced the production of IL-10 and TGF-β by MDSCs from late septic mice.

We detected *S100A9* protein at both IL-10 and TGF-β promoters in MDSCs during late sepsis and levels of *S100A9* bindings at these promoters were significantly reduced after the knockdown of *S100A9* in MDSCs from wild-type mice. Our experiments using *S100A9*-deficient mice further confirmed *S100A9* targeting of IL-10 and TGF-β promoters, as ectopically expressed *S100A9* protein was detected at both promoters. Notably, ectopic expression of *S100A9* in MDSCs from *S100A9*-deficient mice with late sepsis was sufficient to activate IL-10 or TGF-β promoter fused to the luciferase gene. Importantly, in vivo depletion of *Hotairm1* via administration of *Hotairm1* antisense oligonucleotides into wild-type mice significantly reduced *S100A9* protein binding at IL-10 and TGF-β promoters. These findings are significant because we have previously shown that *Hotairm1* induces *S100A9* protein dephosphorylation and accumulation in the nucleus in MDSCs during late sepsis [20,22]. While the current study does not provide mechanistic detail on how *S100A9* regulates IL-10 and TGF-β promoters in MDSCs (e.g., if *S100A9* interacts with transcription co-factors), our results demonstrate that assembly of *S100A9* protein at IL-10 and TGF-β promoters is sufficient to activate their transcription.

MDSCs from wild-type mice and patients with late sepsis produced high amounts of IL-10 and TGF-β upon *ex vivo* stimulation with bacterial LPS. IL-10 and TGF-β are potent immunosuppressive cytokines that mediate and sustain sepsis-induced immunosuppression in animals and humans [27]. IL-10 exerts multiple immunosuppressive effects during sepsis [34]. IL-10 affects both innate and adaptive immune cells. For example, it suppresses monocytes and macrophages by downregulating Major Histocompatibility Complex class II (MHCII) and costimulatory molecules, reducing proinflammatory mediator production such as nitric oxide and increasing macrophage polarization toward the immunosuppressive M2-type macrophage [27,35]. In primed neutrophils, IL-10 can inhibit the production of the proinflammatory cytokines TGF-α and IL-1β as well as Reactive Oxygen Species (ROS) [35,36]. Notably, IL-10 potently inhibits CD4 T cell activation and function [35], while promoting proliferation, survival and anti-inflammatory functions of regulatory T cells in sepsis [37,38], which further augment immunosuppression during sepsis. High IL-10 serum levels correlate with multiple organ dysfunction and mortality in trauma patients [39,40].

TGF-β also is a potent immunosuppressive cytokine that can suppress T cell proliferation and activation [41] and induce macrophage reprogramming to M2-type macrophage [42]. TGF-β also strongly downregulates the proinflammatory functions of activated monocytes and macrophages [41] and induces expansion and activation of Tregs [43,44]. Tregs themselves produce TGF-β and TGF-β can in turn induce the production of IL-10 in Tregs [43], suggesting that TGF-β can form a positive feedback loop to amplify the immunosuppressive effects of MDSCs. Of note, previous studies have reported significant increases in TGF-β levels in septic mice and non-surviving septic patients [46,47]. Whereas other immune cells can produce IL-10 and TGF-β during inflammation and sepsis [27], our findings of increased expression, induced by *S100A9*, of these potent immunosuppressive cytokines in MDSCs during late sepsis further support the immunosuppressive role of MDSCs during sepsis.

MDSCs promote sepsis immunosuppression during late sepsis in mice and humans and persistent increase in MDSCs is associated with elevated risk of secondary infections in chronically ill septic patients [12,18,19]. IL-10 and TGF-β produced by MDSCs during late sepsis can dysregulate the functions of innate and adaptive immune cells, thereby contributing to sepsis immunosuppression [10,27]. We have previously reported that MDSC expansion and sepsis-induced immunosuppression are inhibited in *S100A9*-deficient mice; for example, these mice have significantly lower levels of IL-10 [23]. We also reported that the adaptive transfer of MDSCs from late septic mice into naive mice immediately after sepsis induction significantly increases circulating levels of IL-10 and TGF-β [18]. The current results show that *S100A9* protein increases IL-10 and TGF-β production by MDSCs by activating their promoters. Of note, *Hotairm1* binds to and shuttles *S100A9* protein to the nucleus in MDSCs [20]. In the current study, we used *Hotairm1* antisense oligonucleotides to deplete *Hotairm1* in septic mice. This in vivo targeting of *Hotairm1* significantly reduced IL-10 and TGF-β production by MDSCs, further supporting that *Hotairm1* couples with *S100A9* to promote sepsis-induced immunosuppression by increasing IL-10 and TGF-β levels during late sepsis.

## CONCLUSION

The study highlights the pivotal role of the *S100A9* protein in enhancing the immunosuppressive function of MDSCs during late sepsis. The study demonstrates that nuclear *S100A9* acts as a transcription co-factor, directly binding to the promoters of immunosuppressive cytokines IL-10 and TGF-β, thereby inducing their expression. These cytokines play a crucial role in sustaining the post-sepsis immunosuppressive environment, which is associated with increased susceptibility to secondary infections and poor outcomes in septic patients. Importantly, the depletion of *Hotairm1*, which facilitates *S100A9* nuclear accumulation, significantly reduced IL-10 and TGF-β production, further reinforcing the therapeutic potential of targeting *S100A9* to mitigate sepsis-induced immunosuppression. Overall, these findings suggest that strategies aimed at modulating *S100A9* expression or its interaction with specific promoters could provide novel avenues for improving immune function and outcomes in sepsis.
